# Clinically relevant differences in stress shielding between two short-stemmed femoral prostheses

**DOI:** 10.1007/s00402-025-05975-w

**Published:** 2025-07-09

**Authors:** Felix Werneburg, Annabell Herntrich, Julia Dietz, David Wohlrab, Natalia Gutteck, Delank Karl-Stefan, Alexander Zeh

**Affiliations:** 1 Department of Orthopedic and Trauma Surgery, University Hospital Halle (Saale), Martin Luther University Halle-Wittenberg, Ernst-Grube Str. 40, 06120 Halle, Germany; 2 Faculty of Medicine, Martin Luther University Halle-Wittenberg, Halle (Saale), Germany; 3 Department of Orthopedics and Trauma Surgery, Dölau Hospital, Halle (Saale), Germany

**Keywords:** Short-stem hip prosthesis, Bone mineral density, Stress shielding, Femoral bone remodeling

## Abstract

**Background:**

Short-stemmed endoprostheses were developed to implement proximal load transmission and thus avoid stress-shielding in the proximal femur. Various prosthesis systems have been developed, which are discussed in the literature regarding stress shielding, clinical outcome, and long-term implant stability.

**Methods:**

In this prospective randomized study, 52 patients (27 male, 25 female; average age 60.8 years) with conservatively unsuccessfully treated coxarthrosis were implanted with either a Nanos™ or Optimys™ short-stem prosthesis. Assessment included Gruen-zone based DEXA examinations immediately postoperatively and at one year to evaluate bone mineral density (BMD) and stress shielding, along with clinical outcomes using the Harris Hip Score (HHS). Radiographic measurements included offset (OFF), caput-collum-diaphyseal angle (CCD), leg length (LL), stem migration and inclination, and the occurrence of radiolucent lines (RL), assessed preoperatively, postoperatively, and at 12 months.

**Results:**

DEXA showed differing stress-shielding profiles between stem types, favoring Optimys™ for BMD preservation. The Nanos™ group exhibited significantly greater BMD reduction in Gruen zones 1 (− 10.1%; *p* = 0.001), 4 (− 3.2%; *p* = 0.02), and 7 (− 21.3%; *p* = 0.001), whereas Optimys™ showed a significant decrease only in zone 7 (− 16.2%; *p* = 0.001). Although OFF, CCD, and LL changed significantly within groups postoperatively (*p* < 0.05), no statistically significant differences were found between the two stem designs in the final postoperative measurements (all *p* > 0.05). Stem migration remained clinically irrelevant in both groups. A statistically significant intra-group change was observed only in the Optimys™ group (Nanos™: 1.7 mm, *p* = 0.13; Optimys™: 2.5 mm, *p* = 0.01). Similarly, a small but statistically significant change in stem inclination was observed within both groups (Nanos™: 2.2°, *p* = 0.002; Optimys™: 1.5°, *p* = 0.01). Clinical improvement as measured by the Harris Hip Score (HHS) was excellent in both groups, with no significant differences between systems (Nanos™ pre/post: 52.0 / 98.0; Optimys™ pre/post: 51.6 / 97.0; both *p* < 0.001).

**Conclusions:**

When compared, the Optimys stem demonstrated reduced stress shielding through improved proximal load transmission, resulting in significantly better preservation of bone mineral density in the proximal femur.

**Level of evidence:**

Ib.

## Introduction

Due to an aging population, the number of patients requiring total hip arthroplasty has steadily increased in recent years [1, 2]. Numerous studies have demonstrated that conventional cementless prosthetic stems exhibit excellent longevity and high patient satisfaction. However, there is also a growing demand for total hip arthroplasty among younger and physically active patients [3, 4]. This patient group is statistically at a high risk of requiring at least one revision hip arthroplasty during their lifetime. Consequently, the use of short-stemmed prostheses has gained popularity in recent times [5]. The analysis of the current literature shows that the topic of short-stemmed hip arthroplasty remains in the focus of scientific research. Various aspects and potential advantages of short stem endoprostheses compared to conventional prostheses are in the focus of studies. An emphasis of the scientific work is on the investigation of whether short stem endoprostheses show a reduced stress-shielding of the proximal femur and can therefore be considered as superior to conventional prosthesis stems but also on the potential benefits for obese and elderly patients and in situations with femoral misalignments following post-Perthes disease, post-traumatic deformities or other malpositions of the hip [6].

In addition, it was examined whether there are differences in survival of short- stemmed endoprostheses compared to conventional implants. However, studies on this issue show no inferior results for short-stemmed prostheses [7]. The postulated advantages of short-stemmed prostheses include typically more bone- and muscle-sparing implantation and in addition the avoidance of periprosthetic bone loss (“stress shielding”) due to a more physiological load transfer compared to conventional prosthetic stems [8, 9]. An increased degree of bone loss could raise the risk of aseptic prosthesis loosening and significantly increase the rate and technical demand of necessary revision surgeries. Metaphyseal anchorage is considered a precondition for exclusive metaphyseal load transfer. Preserving metaphyseal bone is advisable to facilitate the transition from a short-stemmed to a conventional prosthesis in cases of aseptic loosening.

Short-stemmed femoral prostheses are intended to facilitate proximal load transfer within the femoral metaphysis in order to reduce stress shielding and preserve metaphyseal bone stock. However, studies evaluating load distribution following the implantation of short-stemmed prostheses have produced inconsistent results regarding the effectiveness of primarily achieving metaphyseal anchorage [10]. Additionally, the extent and clinical relevance of potential bone preservation—as well as the advantages over conventional cementless standard stems—remain subjects of ongoing debate [11–13]. To quantitatively assess load transfer following implantation, dual-energy X-ray absorptiometry (DEXA) has proven to be a precise and reproducible method [14, 15].

This study investigates osseointegration and bone remodeling following implantation of the Nanos™ and Optimys™ prostheses, to determine whether proximal load transfer can be achieved and whether there are discernible differences between the two implant designs.

## Patients and methods

Typical short-stem models used in total hip arthroplasty include the Nanos™ (Smith & Nephew GmbH, Watford, UK) and the Optimys™ (Mathys AG, Bettlach, Switzerland) prosthesis. For optimal load transfer, the Nanos stem prosthesis is designed to anchor in the calcar region, where it binds to the distal lateral cortex to support and compensate for varus loading. The implant features a porous titanium plasma coating to enhance fixation and stability, with additional calcium phosphate to promote osseointegration. The Optimys™ short stem follows the medial curvature of the femur. Its triple-conical design aims to achieve good primary stability, minimizing the risk of postoperative subsidence. Additionally, the titanium plasma spray with calcium phosphate coating is intended to facilitate bone ingrowth on the stem.

This study was approved by the Ethics Committee of Martin Luther University under the registration number 2017-34 (DIMDI 00044265) and reported to the Office for Radiation Protection of the Federal Republic of Germany. A total of 52 patients were evaluated, all of whom underwent total hip arthroplasty (THA) due to advanced hip osteoarthritis (Kellgren-Lawrence grade III–IV) and failed conservative treatment. The patients were randomized into two groups, receiving either the Optimys™ or Nanos™ short-stemmed femoral prostheses. The Optimys™ stems were implanted in 25 patients (10 male, 15 female), with a mean age of 61.3 years (SD 6.4) and an average body mass index (BMI) of 28.4 kg/m² (SD 5.5). The Nanos™ stems were utilized in 27 patients (16 male, 11 female), with a mean age of 60.3 years (SD 6.8) and an average BMI of 27.7 kg/m² (SD 4.0) (Table 1). Patients with previous surgeries in the metaphyseal region or with metabolic bone disorders (e.g., rheumatoid arthritis, osteoporosis, corticosteroid therapy) were excluded from the study.

**Table 1:** Comparison of optimys™ and nanos™ group

Parameter	Optimys™ group (RM monoblock)	Nanos™ group (R3 Implant)	Statistical analysis
Number of patients	25	27	
Gender (M/F)	10/15	16/11	Chi-Square Test, *p* = 0.16
Mean age	61.3 years (SD = 6.4 years)	60.3 years (SD = 6.8 years)	Unpaired tTest, *p* = 0.58
BMI	28.4 (SD = 5.5)	27.7 (SD = 4)	Unpaired t Test, *p* = 0.6
Preoperative CCD	130 degrees (SD = 5°)	131 degrees (SD = 5°)	
Preoperative Harris Hip score	51.6 (SD = 12)	52.0 (SD = 12.3)	Unpaired t Test, *p* = 0.9

*SD* Standard deviation

The prosthetic implantation was performed using a standardized minimally invasive Watson-Jones approach. Postoperatively, all patients were allowed a pain-adapted full weight-bearing. The analgesic regimen followed a standardized protocol, which included intraoperative local infiltration therapy with Ropivacaine and a postoperative oral analgesia regimen (Oxycodone for 4 days, NSAIDs for 10 days, and Novalgin drops as needed). Typically, an outpatient rehabilitation program commenced six weeks after surgery. Native radiological verification of the prosthetic implantation was conducted during the hospital stay.

Subsequent follow-up evaluations for the patients were scheduled at 3 and 12 months postoperatively (Follow-Up 1 (F1): Ø110 days; Follow-Up 2 (FU2): Ø418 days). These evaluations encompassed a clinical assessment, including clinical examination and the assessment of Harris Hip Scores, as well as the identification of any complications, alongside radiological assessment and DEXA measurements.

For all radiological evaluations, measurements were taken for the femoral stem in the anterior-posterior (a.p.) projection, assessing stem migration, stem inclination, the caput-collum-diaphyseal angle (CCD), offset (OFF) and leg length. Stem migration was defined as the distance between the apex of the cone and the tip of the lesser trochanter, while stem inclination was measured as the angle between the tangent to the medial stem contour and the proximal femoral shaft. Leg length was defined as the distance from the ischial line to the tip of the lesser trochanter.

All distance and angle measurements from the radiographs were performed by a single examiner within the PAC-system (version 1.0.0.R812_v20191126_0353 ©2019 Agfa HealthCare N.V., Septestraat 27, B-2640, Mortsel, Belgium). To mitigate the impact of rotational positioning of the proximal femur during a.p. radiography and DEXA, hip joint positioning aids were routinely utilized. The occurrence of periprosthetic radiolucent lines (RL), recorded in the anteroposterior radiographs, was correlated with the Gruen zones [17]. RLs were defined as areas of radiolucency that were at least 1 cm long and 1 mm wide between the prosthesis and the surrounding bone [16]. DEXA scans with Gruen zone analysis were conducted immediately following THA and at FU2.

Statistical analysis was performed using SPSS (IBM SPSS Statistics, Version 19, IBM Company). Significant differences in normally distributed data between different follow-ups were assessed using paired t-tests, while significant differences between the study groups (DEXA) were analyzed using unpaired t-tests. In cases of non-normally distributed data, the Wilcoxon test and Mann-Whitney U test were used. The level of significance was set at *p* < 0.05.

For the case number planning, it is assumed that a difference of 10% of the BMD is relevant as a result of the DEXA. Based on the results of other authors, a standard deviation of 15% was assumed. For all calculations, a power of 80% (β error) and a significance of 5% (α error) were specified. For example, in zone 7, a BMD of 1.3 g/qcm is assumed. The case number calculation results in a number of 27/group. Randomization is carried out by the Institute for Medical Epidemiology, Biometry, and Informatics at MLU (sequentially numbered, sealed, opaque envelopes).

## Results

### Radiological and functional outcomes

A total of 27 femoral stems from the Nanos group and 25 femoral stems from the Optimys group were analyzed, enabling a comprehensive evaluation of the defined clinical and radiological parameters at the follow-up intervals of Ø110 and Ø418 days postoperatively (Table 2).

**Table 2:** Comparative analysis of nanos stems and optimys stems

Parameter	Nanos stem	Optimys stem
Number of implants	27	25
Stem migration (mm)	1.7 (SD = 0.9)	2.5 (*p* = 0.01, SD = 4.5)
Stem tilt (degrees)	2.2 (*p* = 0.002, SD = 9)	1.5 (*p* = 0.01, SD = 2.7)
CCD-angle (degrees)	135 (*p* = 0.02, SD = 4.5)	136 (*p* = 0.02, SD = 5.4)
Leg length discrepancy (mm)	5 (SD = 7)	8.2 (SD = 8.5)
Offset (pre/post (mm))	38.8 (SD = 6,2)/43.3 (SD = 8.1) (*p* < 0.001)	36.4 (SD = 6.3)/42.0 (SD = 6.0) (*p* < 0.001)
Harris hip score (pre/post)	94/98 (SD = 5)	87/97 (SD = 10)

Values in parentheses represent standard deviation (SD). CCD = Caput-Collum-Diaphyseal angle. Only statistically significant values are indicated with p-values; all other values have *p* > 0.05. Values for stem migration and stem tilt represent changes between FU1 and FU2 (paired analysis). P-values for CCD, leg length discrepancy (BL), offset, and HHS refer to comparisons between preoperative measurements and FU2 (Ø418 days postoperatively) using paired t tests

The Nanos stems demonstrated an average migration of 1.7 mm (SD = 0.9), which was not statistically significant between FU1 and FU2 (*p* = 0.13). In contrast, the Optimys stems showed a statistically significant migration increase to 2.5 mm (SD = 4.5) over the same interval (*p* = 0.01).

Analysis of stem inclination revealed statistically significant changes for both prosthesis types within the first 3 months postoperatively. In the Nanos group, inclination changed by 2.2° (SD = 9; *p* = 0.002), and in the Optimys group by 1.5° (SD = 2.7; *p* = 0.01) between the immediate postoperative measurement and FU1 (Ø110 days). No further statistically significant changes in inclination were observed between FU1 and FU2 in either group.

At the second follow-up, the leg length discrepancy was 5 mm (SD = 7) in the Nanos group and 8.2 mm (SD = 8.5) in the Optimys group.

With respect to femoral offset (OFF), both prosthesis types showed a significant postoperative increase. In the Optimys group, the mean offset increased from 36.4 mm (SD = 6.3) preoperatively to 42.0 mm (SD = 6.0) postoperatively (*p* < 0.001). In the Nanos group, the offset increased from 38.8 mm (SD = 6.2) to 43.3 mm (SD = 8.1), also reaching statistical significance (*p* < 0.001). Despite these intra-group changes, no statistically significant difference was observed between the two groups in terms of the absolute postoperative offset (*p* = 0.24).

Functional outcomes, as assessed by the Harris Hip Score (HHS), showed improvement in both groups. The Nanos group increased from a mean HHS of 94 (SD = 5) at follow-up 1 to 98 (SD = 5) at follow-up 2, while the Optimys group improved from 87 (SD = 10) at follow-up 1 to 97 (SD = 10) at follow-up 2. However, no statistically significant differences were found between the Ø110 days and Ø418 days evaluations for either group (*p* > 0.05).

### DEXA results

The DEXA measurements for the Nanos™ prosthesis were evaluated across seven Gruen zones to assess bone mineral density (BMD) immediately postoperatively and at follow-up 2 (Ø418 days postoperatively). For the Nanos™ prosthesis, significant reductions in BMD were observed in Zone 1 (from 0.89 g/cm³ to 0.80 g/cm³, corresponding to a relative decrease of − 10.1%, *p* = 0.001), in Zone 4 (from 2.085 g/cm³ to 2.018 g/cm³, − 3.2%, *p* = 0.02), and in Zone 7 (from 1.50 g/cm³ to 1.182 g/cm³, − 21.3%, *p* = 0.001).

In contrast, the Optimys™ femoral stem demonstrated a statistically significant increase in BMD in Zone 5, from 2.07 g/cm³ to 2.21 g/cm³, corresponding to a relative gain of + 6.8% (*p* = 0.001). A significant reduction in BMD was observed in Zone 7, from 1.48 g/cm³ to 1.24 g/cm³, amounting to a − 16.2% decrease (*p* = 0.001). No statistically significant BMD changes were detected in the remaining Gruen zones. The detailed results of the DEXA measurements for both femoral stems, including relative and absolute changes, are summarized in Table 3.

**Table 3:** Summarization of the bone mineral density (BMD) values (g/cm³) for both the nanos and optimys femoral stems across the seven Gruen zones at follow-up 1 (FU1) and follow-up 2 (FU2).

Gruen Zone	Nanos (FU1) BMD (g/cm³)	Nanos (FU2) BMD (g/cm^3^)	*p*-value	Optimys (FU1) BMD (g/cm³)	Optimys (FU2)	BMD (g/cm^3^)	*p*-value
1	**0.89**	**0.80**	**0.001**		0.85	0.84	> 0.05
2	1.27	1.197	> 0.05		1.30	1.25	> 0.05
3	2.12	2.12	> 0.05		2.12	2.19	> 0.05
4	**2.085**	**2.018**	**0.02**		2.01	2.03	> 0.05
5	1.98	2.034	> 0.05		**2.07**	**2.21**	**0.001**
6	1.68	1.67	> 0.05		1.59	1.60	> 0.05
7	**1.50**	**1.182**	**0.001**		**1.48**	**1.24**	**0.001**

Values in bold indicate statistically significant changes.

## Discussion

In this study, bone remodeling was assessed during postoperative follow-up by analyzing changes in bone mineral density (BMD) using DEXA. This method is widely established as the standard for quantifying periprosthetic bone changes due to its high sensitivity and reproducibility. The clinical importance of preserving proximal femoral bone stock has also been supported by registry data: in a large German registry analysis, Steinbrück et al. (2021) found no statistically significant difference in 5-year implant survival between short-stem and conventional stems in matched patient cohorts (*n* = 17,526 per group) [7].

In addition to biomechanical and radiological assessments, some studies have also examined broader aspects of patient outcomes following implantation of short-stemmed prostheses, such as return to sexual activity and professional life [17, 18]. These investigations found no significant difference between short-stem and conventional designs in terms of time to resumption of sexual activity in patients under 65, or return-to-work intervals [18].

The development of new prostheses stems from the fact that the problems of short-stem endoprosthetics of the hip joint continue to be the focus of implant innovations. There is still intense interest among surgeons in applying this specific type of total hip endoprosthetics. In addition to the potential advantages of preserving bone stock in the proximal femur, surgeons also favor this type of implantation because it is gentle on soft tissue. Short-stem prostheses have shown in studies to be no less effective than conventional stems when used according to the correct indications in terms of long-term stability and safety. Short-stem endoprostheses are not only used in standard situations, but also increasingly in patients with altered anatomy of the proximal femur and, for example, in cases of femoral head necrosis, where they demonstrate excellent results [19].

This study aimed to evaluate and compare bone remodeling and stress shielding between two distinct short-stemmed femoral prostheses, the Nanos™ and Optimys™. The primary focus was to evaluate their ability to achieve optimal proximal load transfer and preserve periprosthetic bone mass, which are critical factors in ensuring long-term implant stability and preventing complications such as aseptic loosening [20].

Our results revealed significant differences in bone mineral density (BMD) changes across different Gruen zones, indicating distinct biomechanical behaviors of these two short-stemmed implant designs, reflecting their potentially differing load transfer capabilities.

### Bone remodeling and stress shielding

The Nanos™ prosthesis demonstrated significant bone loss in Gruen Zones 1 (− 10.1%), 4 (− 3.2%), and 7 (− 21.3%) at FU2 compared to FU1. The Optimys™ prosthesis showed a significant decrease in BMD in Zone 7 (− 16.2%) and a significant increase in Zone 5 (+ 6.8%) over the same interval.

Zones 1 and 7 are critical regions for proximal load transfer. Zone 7, in particular, represents the calcar region [8], which is essential for the distribution of weight during gait in short-stemmed prostheses [21]. The Nanos™ implant demonstrated a pronounced reduction in bone mineral density (BMD) in both Zone 1 and Zone 7. In Zone 1, BMD decreased significantly by approximately 10.1%, while Zone 7 showed an even more substantial reduction of about 21.3% (*p* = 0.001 for both zones). These findings suggest that the Nanos™ prosthesis shows a limited proximal load thereby leading to stress shielding and subsequent reduction of BMD in these critical zones [22, 23].

This result of a postoperative reduction of BMD in zones 1 and 7 with increase in zone 6 is supported by one of our former studies on load-transfer of this implant [10]. In that study, we prospectively analyzed the bone mineral density (BMD) of 25 patients one year after implantation of a Nanos™ stem and observed evidence of stress shielding, with a reduction of BMD in Gruen zones 1 (15%), 2 (5%), and 7 (12%).

In addition, this finding is in accordance with Götze et al. (2010), who found a bone loss of approximately 7% at the calcar region and 6% at the greater trochanter in their study on the osseointegration of the Nanos^®^ prosthesis using DEXA scans [10]. Hypothetically, this could have substantial implications for the long-term durability of the implant, particularly in younger, more active patients, who are more prone to higher mechanical demands on their prostheses.

In contrast, the Optimys™ prosthesis exhibited a more favorable pattern of bone preservation. While there was some postoperative bone loss in Zone 7, with a reduction of approximately 16.2%, the decrease was less pronounced compared to the Nanos™ implant (*p* = 0.001). More importantly, the Optimys™ prosthesis showed no significant decrease in BMD in Zone 1, which may indicate better load transfer to the proximal femur generally and enhanced bone preservation in the femoral neck region.

In general, it can be concluded that for other short-stemmed implants a loss of BMD was reported as high as between 4% and 31% [24] Insofar, the loss of BMD after one year postoperatively is regarded as morderate and currently there is no conclusion regarding the clinical consequence.

### Biomechanical und functional parameters

Both the Nanos™ and Optimys™ prostheses showed no clinically significant differences in biomechanical parameters, including leg length discrepancy (LLD), offset, and caput-collum-diaphyseal (CCD) angle. These findings suggest that, biomechanically, both implants perform similarly in terms of restoring joint function and alignment. Additionally, neither implant demonstrated clinically significant stem migration or inclination during the postoperative period. Both prostheses maintained stable fixation, which is crucial for long-term implant survival. This result supports other studies which demonstrated the potential of reproduction of anatomical (offset, CCD, leg length) with a modern short stem hip design [25].

The finding of reliability of short-stemmed THA is in accordance with other studies [26]. Reichenbacher et al. (2022) reported 87 patients with implanted an unilateral cementless calcar-guided short stem (ANA.NOVA proxy) who received migration analysis with EBRA-FCA. An average migration of 2.0 mm (0.95–3.35) was observed within the first 3 years. Detected migration did not lead to stem loosening, instability, dislocation, or revision surgery in any patient. In that study, a higher risk for subsidence was observed in male and heavyweight patients [27].

The restoration of femoral offset is considered a key factor for optimal biomechanical reconstruction in total hip arthroplasty (THA), influencing muscle tension, joint stability, and range of motion. In this study, both the Nanos™ and Optimys™ groups demonstrated a statistically significant postoperative increase in offset (mean difference: + 4.5 mm and + 5.5 mm, respectively). These findings are in line with previous studies that suggest moderate changes in femoral offset—typically within a range of ± 5 to 6 mm—do not necessarily result in clinically relevant differences in patient-reported outcomes or joint function. Sariali et al. and Mahmood et al. reported that minor increases in offset may improve abductor muscle function, but that such changes do not consistently correlate with superior clinical results [28, 29]. Furthermore, a systematic review by Hall et al. concluded that while restoring offset is important, deviations within a range of ± 5 mm from native anatomy are generally well tolerated and do not significantly impact postoperative mobility or pain levels [30]. Both prosthesis types appear to enable a reproducible and adequate restoration of femoral offset within a range that does not compromise clinical outcomes.

Furthermore, the minimal stem migration and angulation observed in this study are not considered to be indicators of insufficient primary stability or risk factors for reduced long-term stability, as initial migration of a similar magnitude was observed for other short-shaft endoprostheses using precise EBRA-ECA f (single-image X-ray analysis of the femoral component). This initial stem migration was interpreted as initial stabilization and was not found to be a risk factor for revision in a study of 224 short-shaft endoprostheses over an average postoperative period of 84 months. We see a parallel between the minimal significant stem angulation observed in both groups and the minimal stem migration in the Optymys group (Table 2), in that no radiolucent lines relevant to loosening were detected during follow-up and no revision was necessary due to stem loosening [31].

This biomechanical consistency is reflected in the functional outcomes observed. The Harris Hip Score (HHS) is a well-established clinical outcome measure used to assess the functional status of patients after total hip arthroplasty (THA). It evaluates pain, functional capacity, range of motion, and the ability to perform daily activities. In our study, both the Nanos™ and Optimys™ short-stemmed prostheses demonstrated improvements in HHS over time, reflecting positive clinical outcomes in terms of pain relief and functional recovery. In the Nanos™ group, the HHS improved from an average of 94 (SD = 5) at the 3-month follow-up (FU1) to 98 (SD = 5) at the 12-month follow-up (FU2). Similarly, the Optimys™ group showed an improvement from 87 (SD = 10) at FU1 to 97 (SD = 10) at FU2. While both groups exhibited significant improvements in the HHS, no statistically significant differences were observed between the two prosthesis types at either follow-up interval. However, the final result of the HHS after 1 year of follow-up for both groups is considered excellent compared to other studies on clinical outcomes after implantation of a short-stem endoprosthesis, as well as those of conventional prosthesis systems [32].

The clinical relevance of these changes lies in the fact that both groups achieved substantial improvements in function and quality of life, with HHS scores approaching the upper end of the scale. This suggests that both short-stemmed implants were effective in addressing the primary concerns of hip osteoarthritis, namely pain reduction and restoring hip function. Although the Optimys™ group showed a slightly larger improvement in HHS, this difference did not reach statistical significance compared to the Nanos™ group. This finding implies that while the Optimys™ prosthesis might have some advantages in terms of bone preservation, these differences may not be large enough to be considered clinically meaningful in the mid term. Although the HHS improvements in both groups were encouraging, the clinical relevance of these findings may be better understood over a longer period. The potential long-term benefits of improved bone could contribute to sustained or even enhanced functional outcomes over time.

### Clinical relevance and long-term implications

The findings from this study have notable clinical implications, particularly in terms of mid-term bone preservation and implant stability. The Nanos™ prosthesis may be more prone to proximal stress shielding, leading to a greater potential of implant loosening in the long term. This could be particularly problematic for younger and more active patients who place greater mechanical demands on their implants.

In contrast, the Optimys™ prosthesis, which facilitates more effective load transfer to the proximal femur, demonstrated a more favorable remodeling pattern overall. Although a significant reduction in BMD was observed in Zone 7, bone preservation in other proximal regions was maintained. This pattern may support implant longevity and reduce the risk of complications related to periprosthetic bone loss in younger, more active patients who are at increased risk of future revision surgeries [33].

### Strenghts and limitations

This study provides valuable insights into the load transfer and bone preservation capabilities of the Nanos™ and Optimys™ short-stemmed femoral prostheses.

The strengths of the study include the prospective study design and the finding that there were no significant differences in important preoperative characteristic variables of the study population such as age, BMI, COR and CCD. This results in highly comparable study groups.

In addition, the study groups were treated in a standardized intraoperative procedure with regard to the surgical approach, postoperative pain management and physical therapy, which resulted in a high degree of comparability of the clinical results. In addition, the surgeries were performed exclusively by two experienced senior surgeons, so that there was a high degree of standardization.

Furthermore, no statistically significant or clinically relevant differences were found in the parameters characterizing the biomechanics of a THA, such as OFF, CCD and leg-length, so that an influence of these parameters on the postoperative DEXA result can be excluded.

However, there are several limitations that should be acknowledged. The relatively short follow-up period (mean of 418 days) restricts our ability to fully assess the long-term consequences of the observed differences in bone remodeling and stress shielding.

On the other hand, studies on stress shielding following total hip arthroplasty (THA) have shown that the most significant bone remodeling occurs within the first six months postoperatively and tends to plateau after approximately one year. Further changes due to long-term biomechanical adaptation are minor and show no substantial variation [34].

The radiological measurement of stem migration and angulation was not performed by an established method like EBRA. However, the method used in this study was validated and successfully performed for other similar investigations [35]. DEXA scans are widely accepted to investigate the osseointegration of femoral stems [36, 37]. This method is regarded to assess accurately the BMD during the postoperative follow-up which allows conclusions considering the load transfer induced by the femoral implant [38].

When evaluating the results, it is important to note that the number of cases was adjusted to identify statistically significant differences in bone density. For this reason, statistical statements regarding other parameters, such as differences between the groups in terms of biomechanical parameters or clinical outcomes, should only be discussed with this limitation in mind. On the other hand, the focus of this study was on potential differences in stress distribution between these two types of prostheses, so we do not consider this limitation, which in its general form applies to a large number of studies, to be a relevant limitation of the study.

## Conclusion

The Optimys™ stem demonstrated less stress shielding and superior bone preservation in key proximal femur regions compared to the Nanos™ implant. Both implants provided stable fixation and led to improved clinical outcomes. No significant differences were observed in biomechanical parameters. These findings underline the potential of short-stem prostheses as a reliable option in modern total hip arthroplasty.

## Data Availability

The datasets generated and analyzed during the current study are not publicly available due to institutional data protection policies but are available from the corresponding author on reasonable request.
